# Epidemiologic Survey of Japanese Encephalitis Virus Infection, Tibet, China, 2015

**DOI:** 10.3201/eid2306.152115

**Published:** 2017-06

**Authors:** Hui Zhang, Mujeeb Ur Rehman, Kun Li, Houqiang Luo, Yanfang Lan, Fazul Nabi, Lihong Zhang, Muhammad Kashif Iqbal, Suolangsi Zhu, Muhammad Tariq Javed, Yangzom Chamba, Jia Kui Li

**Affiliations:** Huazhong Agricultural University College of Veterinary Medicine, Wuhan, China (H. Zhang, M.U. Rehman, K. Li, H. Luo, Y. Lan, F. Nabi, L. Zhang, M.K. Iqbal, J.K. Li);; Tibet Agriculture and Animal Husbandry College, Linzhi, Tibet, China (S. Zhu, Y. Chamba, J.K. Li);; University of Agriculture, Faisalabad, Pakistan (M.T. Javed)

**Keywords:** epidemiologic survey, Japanese encephalitis virus, viral encephalitis, mosquito vector, transmission, pig farming, swine, amplifying hosts, travel, zoonoses, vector-borne infection, viruses, Tibet, China

## Abstract

We investigated Japanese encephalitis virus (JEV) prevalence in high-altitude regions of Tibet, China, by using standard assays to test mosquitoes, pigs, and humans. Results confirmed that JEV has spread to these areas. Disease prevention and control strategies should be used along with surveillance to limit spread of JEV in high-altitude regions of Tibet.

Japanese encephalitis virus (JEV) is the causative agent of a viral encephalitis that is a major public health threat in most parts of East and Southeast Asia (*1*,*2*). Tibet, China, has been considered a nonepidemic area for JEV because its mean elevation is >3,100 m, and the relatively low temperatures at the elevation do not support JEV transmission between mosquitoes, reservoirs, and amplifying hosts (*2*). However, this situation in Tibet has probably changed due to increased pig farming in the region and increased travel between Tibet and areas where JEV is endemic (*3*–*5*). To test our hypothesis, we tested for JEV in mosquitoes and measured JEV-specific IgM in swine and humans living in a high-altitude region of Tibet.

We conducted the study in Nyingchi District (elevation 3,100 m) in southeastern Tibet. The mean temperature during the study period ranged from 11.3°C to 22.4°C. Swine and humans included in the study had no history of travel to JEV-endemic areas.

To determine whether mosquitoes were infected with JEV, we collected 8,330 mosquitoes (belonging to 9 genera and 4 species) near pig sties and human residences in Nyingchi, Mainling, and Gongbo’gyamda Counties. From those 8,330 mosquitoes, we chose 2,655 JEV vector mosquitoes: 330 (3.96%) *Culex tritaeniorhynchus*, 45 (0.54%) *C. bitaeniorhynchus*, and 2,280 (27.37%) *C. pipiens* mosquitoes. To detect JEV, we used the TIANamp Virus DNA/RNA Kit (TianGen, Beijing, China) with pools of whole-body *Culex* mosquito extracts; we analyzed the samples by reverse transcription PCR (RT-PCR) amplification using the Quant One-Step RT-PCR Kit (TianGen) and primers specific for the JEV nonstructural 1 gene (*6*). Of 11 *C. tritaeniorhynchus* and 69 *C.* *pipiens* mosquito pools, 7 (63.6%) and 2 (2.9%), respectively, were positive for JEV by RT-PCR; the 1 *C. bitaeniorhynchus* mosquito pool was not positive for JEV (Table).

**Table:** Japanese encephalitis virus IgM–positive pigs and humans and Japanese encephalitis virus-positive *Culex* mosquitoes in Nyingchi District, Tibet, China, 2015*

Category, variable	No. IgM positive/no. tested (%)		Total no. positive/total no. tested (%)
	Male pig or person	Female pig or person	
Pigs			
County location			
Nyingchi	3/146 (2.05)	5/100 (5.00)	8/246 (3.25)
Mainling	9/105 (8.57)	6/87 (6.90)	15/192 (7.81)
Gongbo'gyamda	0/9	0/7	0/16
Total	12/260 (4.62)	11/194 (5.67)	23/454 (5.07)
Humans			
County location			
Nyingchi	6/66 (9.09)	11/93 (11.83)	17/159 (10.69)
Mainling	8/58 (13.79)	9/65 (13.85)	17/123 (13.82)
Gongbo’gyamda	1/43 (2.33)	1/39 (2.56)	2/82 (2.44)
Total	15/167 (8.98)	21/197 (10.66)	36/364 (9.89)
Age group, y			
1–23	6/57 (10.53)	7/54 (12.96)	13/111 (11.71)
24–45	7/54 (12.96)	11/80 (13.75)	18/134 (13.43)
>45	2/56 (3.57)	3/63 (4.76)	5/119 (4.20)
Total	15/167 (8.98)	21/197 (10.66)	36/364 (9.89)
*Culex* mosquitoes†			
* C. tritaeniorhynchus*	ND	ND	7/11 (0.636)‡
* C. bitaeniorhynchus*	ND	ND	0/1‡
* C. pipiens*	ND	ND	2/69 (0.029)‡

*Except as indicated, data are no. (%). ND, Not done. †Denominator indicates no. in pool. ‡Maximum likelihood estimates (no. pools positive for JEV by reverse transcription PCR).

To determine the origin of JEV in pigs, we collected a total of 454 serum samples from 1- to 6-month-old pigs from local slaughterhouses. We analyzed the samples for JEV IgM by using a commercial ELISA kit as previously described (*7*). We used the χ^2^ test and SPSS software (SPSS Inc., Chicago, IL, USA) to analyze all data; p<0.05 was considered significant. 

The overall seroprevalence of JEV IgM in the pigs was 5.07%. The percentage of positive samples from Nyingchi County (3.25%) was significantly lower than that from Mainling Country (7.81%); no serum samples from Gongbo'gyamda County were JEV-positive (Table). The difference in seroprevalence of JEV in male (4.62%) and female (5.67%) pigs was not statistically significant.

To determine the prevalence of JEV infection among persons, we collected blood samples from 364 healthy human volunteers residing in the 3 counties and analyzed the samples for JEV IgM by using a commercial ELISA kit as previously described (*8*). JEV seroprevalence was11.71% for the 1- to 23-year-old age group, 13.43% for the 24- to 45-year-old age group, and 4.20% for the >45-year-old age group (Table). Seroprevalence was significantly higher among persons 1–23 or 24–45 years of age, compared with persons >45 years of age, suggesting that 1) younger persons may have greater exposure risks than persons >45 years of age, 2) JEV is a relative new introduction in the area as a result of the changing (i.e., warmer) climate, and 3) younger persons may travel more frequently than older persons to lower elevations where JEV is endemic. An IgG-based survey might identify more JEV disease, even in persons who showed no symptoms of the disease. 

JEV seroprevalence was significantly higher in rural populations (6.87%) compared with urban population (3.02%). The spread of JEV may be increased by amplifying hosts (pigs); thus, the lower prevalence of JEV in urban residents may be associated with a lower number of pig farms in urban areas compared with rural villages in Nyingchi District.

In conclusion, we found that JEV infection is prevalent in a high-altitude region of Tibet that was previously considered to be free from JEV. Factors such as increased tourism (*9*), increased mean summer temperatures, increased movement between the study area and nearby JEV-endemic regions, inadequate public health systems, increased pig farming, increased migration of water birds, and the absence of a compulsory immunization policy may contribute to emergence of this disease (*3*,*4*,*9*,*10*). Disease prevention and control strategies should be used along with surveillance to limit the spread of JEV in high-altitude regions of Tibet.
